# Risk stratification for immune checkpoint inhibitor rechallenge after acute kidney injury: towards a precision medicine framework

**DOI:** 10.3389/fimmu.2026.1889678

**Published:** 2026-07-28

**Authors:** Qiao-Qiao Zhou, Ling Peng

**Affiliations:** 1 Department of Hemodialysis Room, Sichuan Clinical Research Center for Cancer, Sichuan Cancer Hospital & Institute, Sichuan Cancer Center, University of Electronic Science and Technology of China, Chengdu, China; 2 Department of General Internal Medicine, Sichuan Clinical Research Center for Cancer, Sichuan Cancer Hospital & Institute, Sichuan Cancer Center, University of Electronic Science and Technology of China, Chengdu, China

**Keywords:** acute kidney injury, biomarkers, immune checkpoint inhibitors, rechallenge, risk stratification

## Abstract

The decision to rechallenge a patient with immune checkpoint inhibitor−associated acute kidney injury (ICI−AKI) remains one of the most challenging dilemmas in onco−nephrology. Although major guidelines affirm that rechallenge may be considered in selected patients, they uniformly acknowledge a critical gap: no validated tool currently exists to estimate an individual’s risk of recurrence. Reported recurrence rates range from 16.5% to 44%, and the survival benefit of rechallenge is inconsistent across studies. In this review, we synthesize recent evidence on risk factors for ICI−AKI recurrence, including clinical parameters (acute kidney injury (AKI) severity, renal recovery status, extra−renal immune−related adverse events (irAEs), concomitant medications, and immune checkpoint inhibitor (ICI) regimen), pathological findings (acute tubulointerstitial nephritis (ATIN) with Banff scoring, and glomerular diseases), emerging biomarkers (urinary C−X−C motif chemokine ligand 9 (CXCL9)-to-creatinine ratio with an optimal cutoff of 269.5 ng/g, and serum soluble interleukin−2 receptor alpha (sIL−2Rα) with a cutoff of ≥1.75× the upper limit of normal (ULN)), genetic markers (the propionyl−CoA carboxylase subunit alpha (*PCCA*) variant rs16957301), and acute kidney disease (AKD). We translate this evidence into a practical, stepwise risk stratification framework that classifies patients into low−, intermediate−, and high−risk tiers through the sequential integration of clinical, pathological, and biomarker information. The biomarker cutoffs included in this framework—derived from diagnostic studies—are hypothesis−generating in the context of recurrence prediction and require prospective validation before clinical application. For each tier, we provide corresponding recommendations regarding rechallenge decisions, monitoring intensity, and prophylactic immunosuppression, while acknowledging the limited evidence supporting prophylactic corticosteroids in this setting. Finally, we discuss current controversies—including the optimal timing of rechallenge, racial differences that challenge model generalizability, and the unresolved role of prophylactic glucocorticoids—and outline future research priorities organized around clinical validation, biomarker development, mechanistic exploration, and integration of emerging technologies. This framework is designed for immediate clinical application across diverse settings, ranging from resource−limited primary hospitals to tertiary centers. By transforming empirical decision−making into evidence−based, individualized risk assessment, this review aims to guide precision rechallenge management for patients recovering from ICI−AKI.

## 1 Introduction: the dilemma and opportunity

Immune checkpoint inhibitors (ICIs) have revolutionized cancer treatment by restoring T-cell-mediated antitumor immunity. This approach offers significant survival benefits (1–3). However, this therapeutic success is frequently accompanied by immune-related adverse events (irAEs) (4, 5). Among these, ICI-associated acute kidney injury (ICI-AKI) is reported in approximately 1–5% of treated patients (6, 7), although AKI from any cause affects up to 16–18.2% of this population, as documented in multiple cohort studies and meta-analyses (8–13).

The decision to resume immunotherapy after an episode of ICI-AKI—termed “rechallenge”—presents a core clinical dilemma. Withholding ICIs may permit unchecked tumor progression. Indeed, a Canadian population-based cohort study (n = 16,425) demonstrated that patients rechallenged with ICIs within 6 months of AKI experienced significantly improved overall survival compared with those switched to non-ICI therapies (adjusted hazard ratio (HR) 0.38, 95% CI 0.22–0.67; P < 0.001) (14). In contrast, this survival advantage is not uniformly observed. An international multicenter study of 429 patients with ICI-AKI identified no significant survival difference between those who were rechallenged and those who were not (8), suggesting that the net oncologic benefit is likely contingent upon individual patient characteristics. The challenge of ICI rechallenge decisions is not unique to the post-AKI setting; it has been increasingly recognized across various cancer types and clinical scenarios. In hepatocellular carcinoma (HCC), for example, rechallenge after initial ICI discontinuation due to tumor progression or immune-related adverse events has emerged as a feasible salvage strategy, with multiple retrospective studies and case series reporting variable but notable antitumor activity (15, 16). These studies underscore that the rechallenge decision—regardless of the specific indication—inherently involves balancing uncertain oncologic benefit against potential risks. Compounding this uncertainty, rechallenge carries a substantial risk of recurrent kidney injury, with reported incidence rates ranging from 16.5% to 44% across large cohort studies (8, 14). Consequently, the confluence of uncertain oncologic benefit and considerable renal risk underscores an urgent unmet need: a precision medicine framework to guide individualized rechallenge decisions.

Authoritative reviews have established foundational principles for the diagnosis and management of ICI-AKI, affirming that kidney biopsy remains the diagnostic gold standard, that glucocorticoids constitute first-line therapy, and that rechallenge may be considered in selected patients (6, 7, 17, 18). Nevertheless, these consensus documents collectively reveal a critical gap: the absence of tools to quantify individual recurrence risk. This ambiguity stems from the substantial heterogeneity of ICI-AKI risk, which arises from an interplay of clinical (19, 20), pathological (7, 21–24), genomic (25), and microenvironmental factors (26–30). Extending beyond the acute injury phase, the subacute spectrum—termed acute kidney disease (AKD)—represents a frequent complication following ICI therapy, a concept well established in nephrology (31). This prolonged window of renal risk further underscores the need for a dynamic assessment that transcends the initial AKI event. Such assessment is particularly urgent given racial differences that challenge model generalizability: while the propionyl-CoA carboxylase subunit alpha (*PCCA*) risk variant has only been validated in White populations (25), Asian race is independently associated with higher ICI-AKI risk (32).

To address this gap, this review systematically evaluates the multidimensional evidence informing rechallenge risk stratification after ICI-AKI. Synthesizing these data, we propose, for the first time, an integrative three-step framework that sequentially incorporates clinical assessment, pathological evaluation, and molecular biomarkers to guide precision-based rechallenge decisions across diverse clinical settings. Comprehensive reviews of the broader epidemiology, diagnosis, and management of ICI-associated nephrotoxicity are available elsewhere (33, 34), including a recent overview by Zhou et al. (20) that synthesized ICI-AKI epidemiology, mechanisms, and general management. However, the specific question of which patients can safely be rechallenged after ICI-AKI—identified as a critical gap by all major guidelines—was not the focus of those works. The present review focuses exclusively on this question.

## 2 Multidimensional evidence base for risk stratification

### 2.1 Clinical parameters and risk factors

Baseline renal impairment is a key predictor of ICI-AKI. Reduced baseline estimated glomerular filtration rate (eGFR) has been identified as an independent risk factor for ICI-AKI across multiple multicenter cohorts (8, 14, 35, 36). In one such study, lower baseline eGFR conferred an adjusted odds ratio (OR) of 1.99 (95% CI 1.43–2.76) for each 30 mL/min/1.73 m² decline, and chronic kidney disease (CKD) (eGFR < 60 mL/min/1.73 m²) was significantly more common among patients with ICI-AKI than among controls (32% vs 20%, P = 0.01) (36). Baseline CKD significantly increases ICI-AKI risk, with pooled adjusted ORs ranging from 1.86 to 2.90 in meta-analyses (10, 19). A pooled OR of 2.90 (95% CI 1.65–5.11) for pre-existing CKD places it among the strongest clinical predictors (19).

Extra-renal irAEs constitute another critical harbinger of ICI-AKI, with multiple studies consistently demonstrating an independent association with heightened renal risk (8, 10, 19, 37, 38). The presence of extra-renal irAEs conferred a pooled OR of 2.53 (95% CI 1.79–3.56) for ICI-AKI in one meta-analysis (10), and an adjusted OR of 2.45 (95% CI 1.62–3.72) was observed in an international multicenter study (8). This association suggests that extra-renal irAEs may signal a state of systemic immune hyperactivation that can extend to renal injury (8, 38). Strikingly, the association between extra-renal irAEs and AKI remained significant after multivariable adjustment (OR 2.82, 95% CI 1.45–5.48), underscoring its independence from potential confounders (12).

Concomitant medications represent well-established and potentially modifiable risk factors. Proton pump inhibitors (PPI) have been investigated extensively, although confounding by indication may partially account for the observed association (39–44). A Danish population-based cohort study found that the association between PPI use and AKI attenuated considerably after propensity score weighting (weighted HR 1.06, 95% CI 0.93–1.21), suggesting that earlier estimates may have been confounded by underlying comorbidities (41). Conversely, a nested case-control study reported an adjusted OR of 2.62 (95% CI 1.75–3.93) for concurrent PPI and ICI use compared with neither exposure (43). Beyond PPI, other agents—including non-steroidal anti-inflammatory drugs (NSAIDs), diuretics, and renin-angiotensin system (RAS) inhibitors—also elevate risk, as confirmed by meta-analyses and pharmacovigilance data (14, 19, 40, 45–49). Pooled ORs of 2.61 (95% CI 1.90–3.57) for NSAIDs, 1.78 (95% CI 1.32–2.40) for diuretics, and 1.76 (95% CI 1.15–2.68) for RAS inhibitors have been reported (19). Consistent with these findings, an analysis of the U.S. Food and Drug Administration (FDA) pharmacovigilance database yielded a reporting odds ratio (ROR) of 2.10 (95% CI 1.91–2.31) for concurrent PPI exposure, whereas NSAIDs and diuretics showed RORs of 3.06 and 2.82, respectively (48). Strikingly, the addition of ICIs to chemotherapy is associated with a nearly three-fold higher AKI risk compared with chemotherapy alone (RR 2.89) (50).

The specific ICI regimen also influences risk: combination therapy with anti−cytotoxic T lymphocyte−associated antigen 4 (anti-CTLA-4) and anti−programmed cell death protein 1 (anti-PD-1) agents carries higher nephrotoxic potential than monotherapy, with combination therapy consistently identified as an independent risk factor (10, 19, 32, 36, 51–53). In a pooled analysis of biopsy-proven cases, all 19 patients receiving dual ICI blockade developed Kidney Disease: Improving Global Outcomes (KDIGO) stage 3 AKI, compared with 29 of 60 patients (48%) receiving monotherapy (P < 0.001) (53). A meta-analysis reported a pooled OR of 2.45 (95% CI 1.40–4.31) for combination therapy versus monotherapy (19). Furthermore, in biopsy-proven cohorts, combination therapy is associated with more severe AKI and lower odds of complete renal recovery (53, 54). Illustrating this point, a case of nephrotic syndrome with AKI was described following a single cycle of nivolumab plus ipilimumab, with biopsy revealing minimal change disease superimposed on acute interstitial nephritis (54).

Additional risk factors include diabetes mellitus (10, 14), hypertension (12, 14), Asian race (32), and genitourinary malignancies—bladder and kidney cancer being associated with the highest AKI risk compared with melanoma (14, 55). In a Canadian population-based cohort, genitourinary malignancy was associated with heightened AKI risk, whereas a single-institution analysis identified Asian race as an independent predictor (14, 32). Of note, in patients with advanced CKD, ICI therapy was not associated with increased AKI risk when compared with other antineoplastic agents (56). A retrospective cohort study comparing 91 patients with advanced CKD (eGFR < 30 mL/min/1.73 m²) receiving ICIs versus those receiving either nephrotoxic or non-nephrotoxic antineoplastic therapies found no significant differences in AKI rates (17.5% vs 17.6% vs 20%, P = 0.87) or new-onset kidney failure (56).

**Table 1** summarizes the major risk factors for ICI-AKI derived from meta-analyses and large cohort studies, encompassing baseline characteristics, ICI regimen, concomitant medications, treatment-related factors, and extra-renal manifestations.

**Table 1:** Major risk factors for ICI-AKI.

Risk factor	Effect size (95% CI)	Study type/data source	Reference
Baseline characteristics			
Chronic kidney disease (CKD)	OR 1.86–2.90	Meta-analysis	(10, 19)
Diabetes mellitus	OR 1.26–1.28	Meta-analysis	(10, 14)
Hypertension	OR 4.3 (1.8–6.1); aHR 1.19 (1.05–1.35)	Multicenter cohort; Population-based cohort	(12, 14)
Asian race (vs White)	aHR 4.18–4.39 (1.09–16.04)	Single-center cohort	(32)
Lower baseline eGFR (per 30 mL/min/1.73 m² decline)	OR 1.99 (1.43–2.76)	Multicenter cohort	(36)
ICI regimen			
Combination therapy (anti-CTLA-4 + anti-PD-1/PD-L1, vs monotherapy)	OR 2.45 (1.40–4.31)	Meta-analysis	(19)
Concomitant medications			
Proton pump inhibitor (PPI)	OR 2.07–2.23	Meta-analysis	(19, 48)
Non-steroidal anti-inflammatory drugs (NSAIDs)	OR 2.61 (1.90–3.57)	Meta-analysis	(19)
Diuretics	OR 1.78 (1.32–2.40)	Meta-analysis	(19)
RAS inhibitor	OR 1.76 (1.15–2.68)	Meta-analysis	(19)
Treatment-related			
ICI + chemotherapy (vs chemotherapy alone)	RR 2.89 (1.37–6.10)	Meta-analysis of RCTs	(50)
Extra-renal manifestations			
Extra-renal irAE	OR 2.34–2.53	Meta-analysis	(10, 19)

OR, odds ratio; RR, risk ratio; aHR, adjusted hazard ratio; CI, confidence interval; eGFR, estimated glomerular filtration rate; RAS, renin-angiotensin system; irAE, immune-related adverse event. The association for Asian race is based on a small sample (n=12 Asian patients) and requires further validation. Effect sizes shown as ranges (e.g., OR 1.86–2.90) represent pooled estimates from multiple meta-analyses.

### 2.2 Pathological subtypes and prognostic implications

Kidney biopsy remains the gold standard for definitive diagnosis and prognostic assessment of ICI-AKI (6, 7).

Acute tubulointerstitial nephritis (ATIN) represents the predominant histopathological phenotype, accounting for 82-93% of biopsy-confirmed cases across multiple case series and cohort studies (6–8, 34, 36, 57–60). In an international multicenter study of 151 biopsied patients, 125 (82.7%) had ATIN, with a median time from ICI initiation to AKI of 16 weeks (8). An even higher proportion—93%—was reported in another multicenter cohort (36). A diagnosis of ATIN is associated with more favorable renal outcomes, particularly when immunosuppression is initiated promptly (8, 61). Corticosteroid treatment within 14 days of AKI diagnosis was associated with higher odds of renal recovery (adjusted OR 2.64, 95% CI 1.58–4.41), and early initiation within 3 days conferred additional benefit (adjusted OR 2.09, 95% CI 1.16–3.79) (8). In a single−center cohort of biopsy−proven ICI−ATIN, although most patients responded to corticosteroid therapy, complete renal recovery was achieved in less than half of cases (62). Interestingly, patients with a clinical diagnosis of “estimated AIN” (eAIN)—made without confirmatory biopsy—paradoxically exhibit lower one-year mortality despite presenting with more severe AKI, a finding that suggests that eAIN may serve as a surrogate marker for a robust systemic antitumor immune response (63). Specifically, patients with eAIN had significantly lower mortality than those with AKI from other causes (adjusted HR 0.44, 95% CI 0.21–0.93), consistent with the hypothesis that eAIN reflects effective immune activation against the tumor (63).

Although less common than ATIN, ICI−associated glomerular diseases encompass a diverse spectrum of histopathological entities, including pauci−immune glomerulonephritis (GN) and renal vasculitis, podocytopathies (minimal change disease and focal segmental glomerulosclerosis), C3 GN, IgA nephropathy, membranous nephropathy, anti−glomerular basement membrane disease, and thrombotic microangiopathy (7, 64). In a systematic review of 45 biopsy−proven cases, pauci−immune GN/vasculitis accounted for 27% of glomerular lesions, podocytopathies for 24%, and C3 GN for 11%, with concomitant ATIN present in 41% of cases (64). The clinical presentation of these glomerular entities differs from the typical ATIN phenotype: patients often present with nephrotic−range proteinuria, hematuria, or rapidly progressive kidney function decline, rather than the relatively bland urine sediment and subacute creatinine rise characteristic of ATIN (64, 65).

Importantly, the prognosis and management of ICI−associated glomerular diseases differ substantially from ATIN. Whereas ATIN generally shows favorable response to corticosteroid monotherapy—with complete recovery in approximately 40% of biopsy−proven cases (36, 53)—glomerular pathologies often follow a more severe clinical course and may require alternative immunosuppressive strategies. In the systematic review by Kitchlu et al., only 31% of patients with glomerular disease achieved complete renal recovery and 42% achieved partial recovery despite corticosteroid treatment in 98% of cases, with 19% remaining dialysis−dependent (64). Among patients with pauci−immune GN or vasculitis, rituximab or cyclophosphamide has been used with variable success, whereas patients with podocytopathies may respond to corticosteroids alone but carry a risk of relapse upon ICI rechallenge (64–66). In a recent series, patients with ICI−induced glomerulonephritis receiving rituximab had a shorter median corticosteroid duration (2.5 versus 8 weeks) and better proteinuria response compared with those receiving corticosteroids alone (66). Rituximab has also been reported to successfully treat ICI−associated multiorgan vasculitis, with sustained cancer remission in a case of anti−PD−L1−related vasculitis (67).

These differences in clinical presentation, prognosis, and treatment response have direct implications for rechallenge decisions. For patients with biopsy−proven isolated ATIN and complete renal recovery, rechallenge may be considered after careful risk−benefit assessment. By contrast, for patients with crescentic GN, vasculitis, or other severe glomerular pathologies, rechallenge is generally discouraged regardless of the degree of renal recovery, as recurrent injury may be more severe and less responsive to corticosteroids (7, 64, 68). For patients with podocytopathies who achieve complete remission, rechallenge may be considered with caution and close monitoring for proteinuria recurrence, though data are limited to case reports (64). These distinctions inform the risk stratification criteria for patients with glomerular pathology, as outlined in Section 3.1. Standardized grading of interstitial inflammation using modified Banff criteria (*i*-score 0−3) provides quantifiable measures that further refine risk stratification (69, 70).

### 2.3 Genomic markers and genetic susceptibility

Emerging evidence points to a genetic contribution to ICI-AKI susceptibility. In a real-world cohort study using the All of Us database (n = 414 ICI-treated patients), the *PCCA* variant rs16957301 (TC/CC genotype) was associated with significantly increased ICI-AKI risk in patients of European ancestry (OR 2.53–2.56; Bonferroni-corrected P = 0.047) (25). Encouragingly, this variant did not confer significant AKI risk in the general population without ICI exposure, suggesting a genotype-specific interaction with ICI therapy (25). However, the genetic architecture underlying ICI-AKI exhibits racial heterogeneity. Although Asian race is independently associated with higher ICI-AKI risk (32), the *PCCA* risk variant has only been validated in White populations. This disparity highlights the critical need for ancestry-specific genetic studies (32).

Broader genomic interrogation may uncover additional susceptibility loci beyond germline variants. For instance, an expression quantitative trait locus (eQTL) mapping study at single-cell resolution across 1,073 individuals identified a T cell–specific *HLA-DQA1* eQTL (rs3104371) with strongest effects in cytotoxic CD8+ T cells—a finding with potential relevance for ICI-AKI susceptibility given the central role of T cells in its pathogenesis (71).

### 2.4 Microenvironmental characteristics and liquid biopsy markers

#### 2.4.1 Diagnostic biomarkers for ICI-AKI

Urinary biomarkers have emerged as promising non-invasive diagnostic tools for ICI-AKI. Among these, urinary C-X-C motif chemokine ligand 9 (CXCL9) demonstrates robust diagnostic performance, with AUC values ranging from 0.84 to 0.94 across studies (26–28, 72, 73). In the primary derivation cohort, an optimal cutoff of 269.5 ng/g (urinary CXCL9-to-creatinine ratio) yielded 82% sensitivity and 85% specificity for distinguishing biopsy-proven ICI-AIN from other AKI causes (26), while a cutoff of 248.1 ng/g was reported in a subsequent multicenter validation (72). Urinary CXCL9 outperformed Tumor Necrosis Factor-alpha (TNF-α) (AUC 0.83 vs. 0.63) (28). Other urinary cytokines, including TNF-α, Interleukin-2 (IL-2), and IL-10, have also shown promise (29, 74–76). Urinary T cells exhibit clonotypic identity with kidney T cell infiltrates, supporting non-invasive monitoring (77). Markers of tubular injury (Neutrophil Gelatinase-Associated Lipocalin, Kidney Injury Molecule-1) may enable detection of subclinical damage prior to serum creatinine elevation (76).

Blood-based biomarkers offer complementary systemic perspectives. Elevated soluble interleukin-2 receptor alpha (sIL-2Rα; cutoff ≥1.75 × upper limit of normal (ULN)) achieved AUC >0.96 for diagnosing ICI-nephritis (30). Dynamic changes in peripheral CD8+ T cell subsets correlate with irAE severity; under dual PD-1/CTLA-4 blockade, patients with severe irAEs exhibited distinct CD8+ T cell alterations compared with those with mild irAEs (78). Because ICI-associated ATIN is characterized by CD8+ T-cell predominant interstitial infiltration (7, 21), tracking peripheral CD8+ T cell dynamics may offer a non-invasive window into kidney-specific immune activation.

Although less validated for isolated ICI-AKI, composite cytokine scores from overall irAE studies offer insights for renal risk stratification. In a prospective cohort of 131 skin cancer patients, a score integrating IL-7, IL-1RA, and CXCL13 independently predicted overall irAE risk (AUC 0.710; multivariable HR 1.41, 95% CI 1.07–1.85; P = 0.014) (79). Given that ATIN, the predominant phenotype of ICI-AKI, is driven by similar T-cell-mediated pathways, these findings provide important candidate directions for developing ICI-AKI-specific biomarkers.

#### 2.4.2 Biomarkers in the context of rechallenge: from diagnosis to prediction

A critical distinction must be emphasized: all biomarkers discussed above have been validated for the diagnosis of acute ICI-AKI, and their utility in predicting recurrent nephrotoxicity following ICI rechallenge has not been established. The studies cited in Section 2.4.1 evaluated these markers at the time of AKI presentation, not at renal recovery or immediately prior to rechallenge. Whether biomarker levels measured during the recovery phase—or their dynamic trajectories—can identify patients at increased risk of recurrence upon rechallenge remains unknown. Therefore, the inclusion of these biomarkers in the risk stratification framework proposed in Section 3.1 is hypothesis-generating rather than evidence-based, and their clinical application for this purpose awaits prospective validation.

Among the urinary markers, CXCL9-to-creatinine ratio has the most robust diagnostic data (26, 72, 73), and its threshold of 269.5 ng/g might serve as a candidate cutoff for heightened recurrence risk if future studies validate that elevated CXCL9 at the time of renal recovery predicts recurrent AKI. Similarly, the serum sIL-2Rα cutoff of ≥1.75×ULN (30) may identify patients with ongoing subclinical immune activation, but its predictive value for recurrence is entirely hypothetical. Other markers—including urinary TNF-α, the IL-5/Fas signature, and composite cytokine scores—have shown diagnostic promise (29, 74, 79) but require dedicated prospective evaluation for their predictive utility in the rechallenge setting.

Beyond soluble factors, emerging cellular and imaging markers are gaining research attention. Tissue-resident memory T (Trm) cells can persist in the kidney following initial injury and orchestrate recurrent nephritis upon rechallenge in murine models, with similar residency markers identified in human interstitial nephritis (80). Whether Trm cell abundance in biopsy specimens or urine correlates with recurrence risk is an important research question. CD163+ alternatively activated macrophages have been detected in ICI-AIN, with infiltration correlating inversely with initial eGFR and positively with renal improvement at three months (81), suggesting a protective or reparative role. Preliminary data also suggest that fluorine-18 fluorodeoxyglucose positron emission tomography (F18-FDG PET) may detect ICI-associated AKI through increased renal fluorodeoxyglucose (FDG) uptake (82). Both modalities remain experimental and require prospective validation before clinical application.

**Table 2** summarizes the current evidence status of each biomarker class, distinguishing their validated diagnostic role from their hypothetical predictive role.

**Table 2:** Biomarker evidence status: diagnostic vs. predictive applications.

Biomarker	Validated diagnostic performance	Evidence for predicting recurrence after rechallenge	Recommended use
Urinary CXCL9−to−creatinine ratio	AUC 0.84–0.94; cutoff 269.5 ng/g (26, 72, 73)	None	Research only (hypothesis−generating)
Serum sIL−2Rα	AUC >0.96; cutoff ≥1.75×ULN (30)	None	Research only (hypothesis−generating)
Urinary TNF−α	AUC 0.83 (28)	None	Research only
Urinary IL−5/Fas signature	AUC 0.94 (74)	None	Exploratory research
Composite cytokine score	AUC 0.710 for overall irAE risk (79)	None	Exploratory research
Trm cells	Not validated for diagnosis	Preclinical evidence only (80)	Mechanistic research
CD163+ macrophages/F18−FDG PET	Not validated for diagnosis	Hypothetical (81, 82)	Exploratory research

AUC (area under the curve) 0.94 was reported in a discovery cohort and requires prospective validation.

ULN, upper limit of normal; Trm, tissue−resident memory T; PET, positron emission tomography; FDG, fluorodeoxyglucose; AUC, area under the curve; CXCL9, C−X−C motif chemokine ligand 9; sIL−2Rα, soluble interleukin−2 receptor alpha; TNF−α, tumor necrosis factor−alpha; IL−5, interleukin−5; Fas, Fas cell surface death receptor; CD163, cluster of differentiation 163; ICI−AKI, immune checkpoint inhibitor−associated acute kidney injury. All diagnostic performance data are derived from studies of acute ICI−AKI diagnosis; none of these markers have been validated for predicting recurrence upon rechallenge.

## 3 Constructing a stepwise risk stratification model

### 3.1 Definition criteria for three-tier risk stratification

**Figure 1:**
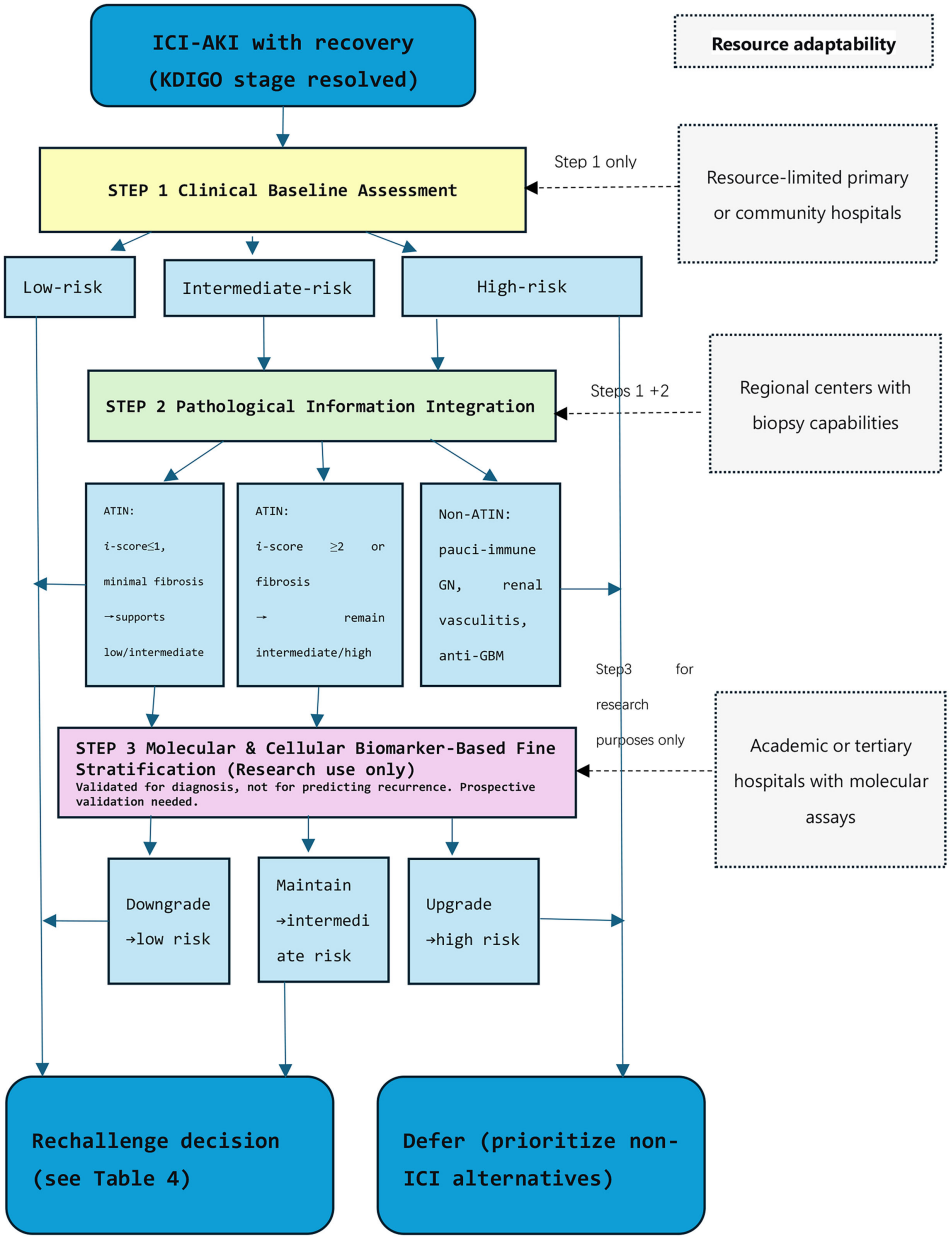
Stepwise risk assessment framework for ICI rechallenge after ICI-AKI. This three-step framework integrates clinical (Step 1), pathological (Step 2), and molecular (Step 3) domains to guide risk stratification. Step 3 is reserved for research purposes only, as the biomarkers included have been validated for diagnosis of acute ICI-AKI, not for predicting recurrence upon rechallenge. For high-risk patients without atypical features, rechallenge is deferred in favor of non-ICI alternatives. For those with atypical features (e.g., inconclusive presentation, suspected glomerular pathology, or steroid resistance), Steps 2 and, where applicable, Step 3 for research purposes provide further refinement. Resource adaptation: Step 1 alone for primary/community hospitals; Steps 1 + 2 for regional centers with biopsy; Step 3 for research purposes only in academic/tertiary centers with molecular assays. Abbreviations: ATIN, acute tubulointerstitial nephritis; GN, glomerulonephritis; anti-GBM, anti-glomerular basement membrane disease.

The evidence reviewed above indicates that the risk of recurrent ICI-AKI is multifactorial and cannot be reliably estimated by any single parameter. We propose a three-step approach that sequentially integrates clinical, pathological, and molecular biomarker information to classify patients into low-, intermediate-, and high-risk tiers (**Figure 1**). This framework is designed to be adaptable across diverse clinical settings: community hospitals may primarily apply Step 1 (Clinical Baseline Assessment); regional centers with biopsy capabilities may integrate Step 2 (Pathological Information Integration); and academic centers with advanced molecular assays may apply Step 3 for research purposes, though its clinical utility in predicting recurrence remains investigational.

Several important limitations must be acknowledged at the outset. First, the proposed risk strata are derived from risk factors associated with initial ICI−AKI incidence, and their predictive value for recurrence upon rechallenge has not been directly established in prospective cohorts. Second, the criteria defined below are intended to guide clinical reasoning and research design, not to serve as a validated clinical prediction rule. Third, quantitative weights for individual risk factors are unavailable, as no prospective study has derived a multivariable model specifically for recurrence risk. **Table 3** should therefore be interpreted as a framework for structured decision−making rather than a fixed algorithm.

**Table 3:** Three-tier risk stratification model for ICI rechallenge after ICI-AKI.

Risk tier	Core criteria	Modifying factors	Evidence/remarks	Recommended action
Low	ALL of the following: (1) Isolated KDIGO stage 1 AKI with renal recovery (SCr <1.5× baseline within 90 days); (2) No extra-renal irAEs; (3) No high-risk medications (PPI, NSAIDs, combination ICI therapy); (4) Non-Asian race	(a) Banff i-core ≤1; (b) Urinary CXCL9-to-creatinine ratio <269.5 ng/g*; (c) sIL-2Rα <1.75×ULN*	Core criteria: derived from meta−analyses and large cohorts (8, 10, 19). Asian race: single−center study, n=12 (32); predictive value for recurrence unknown. Modifying factors: Banff i-score from biopsy cohorts (61); biomarkers diagnostic−only (26, 30)	Rechallenge can proceed
Intermediate	ANY of the following: (1) KDIGO stage 2 AKI or incomplete recovery; (2) History of extra-renal irAEs; (3) Modifiable risk factors (baseline CKD, PPI/NSAIDs use); (4) Other clinical risk factors (Asian race, diabetes, hypertension, genitourinary malignancy)	(a) Banff i-score 1–2; (b) Mildly elevated urinary CXCL9-to-creatinine ratio (e.g., 25–200 ng/g)*; (c) Delayed glucocorticoid initiation (>3 days after AKI diagnosis)	Core criteria: extra−renal irAEs (pooled OR 2.53 from meta−analysis) (10); CKD (pooled OR 2.90) (19); genitourinary malignancy (14). Modifying factors: Banff i-score 1–2 (61); CXCL9 range post−hoc, requires validation; delayed steroids from Gupta et al. (8)	Rechallenge may be considered with caution
High	ANY of the following: (1) KDIGO stage 3 AKI or dialysis requirement; (2) Multi-organ irAEs (≥2 organ systems); (3) No response to glucocorticoids (no improvement after 3–7 days of prednisone 0.5–1 mg/kg/day)	(a) Banff i-score ≥2 or concomitant glomerular pathology; (b) PCCA rs16957301 risk genotype; (c) Urinary CXCL9-to-creatinine ratio ≥269.5 ng/g*; (d) sIL-2Rα ≥1.75×ULN*	Core criteria: KDIGO stage 3 associated with initial AKI (8, 36), predictive value for recurrence unproven; multi−organ irAEs from Gupta et al. (8). Modifying factors: glomerular pathology from Kitchlu et al. (64); PCCA European−ancestry only (25); biomarkers diagnostic−only (26, 30)	Rechallenge generally deferred

AKI, acute kidney injury; SCr, serum creatinine; irAE, immune-related adverse event; PPI, proton pump inhibitor; NSAIDs, non-steroidal anti-inflammatory drugs; ICI, immune checkpoint inhibitor; CKD, chronic kidney disease; ULN, upper limit of normal; sIL-2Rα, soluble interleukin-2 receptor alpha.

*These biomarkers have been validated for the diagnosis of acute ICI−AKI, not for predicting recurrence after rechallenge. The 25–200 ng/g range for intermediate risk is derived from post−hoc analysis and requires prospective validation. Their inclusion as modifying factors is hypothesis−generating.

Throughout this framework, AKI severity is defined according to the Kidney Disease: Improving Global Outcomes (KDIGO) criteria: stage 1, 1.5–1.9 × baseline serum creatinine (SCr); stage 2, 2.0–2.9 × baseline; and stage 3, ≥3.0 × baseline or initiation of renal replacement therapy (RRT) (6, 7, 83, 84). Although the Common Terminology Criteria for Adverse Events (CTCAE) (85) has been employed in some oncology trial contexts, we follow the clinical practice guidelines for ICI-AKI (6, 7, 84) and adopt KDIGO criteria for risk stratification, as these provide a more granular and clinically validated framework for assessing renal recovery and guiding rechallenge decisions.

To address the inherent complexity of clinical decision−making and the absence of quantitative weight estimates, we propose a hierarchical risk factor classification. Risk factors are categorized into decisive factors (Level 1), which when present alone may be sufficient to classify a patient into a given risk tier and include histopathological findings with established prognostic implications (e.g., crescentic glomerulonephritis, vasculitis), multi−organ irAEs, and glucocorticoid resistance; and modifying factors (Level 2), which influence risk but require corroboration with other clinical or pathological information before prompting reclassification and include AKI severity, extra−renal irAEs, concomitant medications, and demographic factors.

The low-risk category requires fulfillment of all core criteria: (1) isolated mild AKI (KDIGO stage 1) with complete renal recovery, defined as SCr < 1.5 × baseline within 90 days (84); (2) absence of extra-renal irAEs; (3) no exposure to high-risk medications, including PPI, NSAIDs, and combination ICI therapy; and (4) non-Asian race (see caution below). Modifying factors that further support low−risk classification include: (a) minimal or no interstitial inflammation on biopsy (Banff *i*-score ≤ 1) (61); (b) normal or low urinary CXCL9-to-creatinine ratio (< 269.5 ng/g) (26); and (c) normal sIL-2Rα level (<1.75×ULN) (30). The biomarker−based criteria, however, are derived from diagnostic studies and have not been validated for predicting recurrence; their inclusion is hypothesis−generating.

The intermediate-risk category requires the presence of any core criterion: (1) moderate AKI (KDIGO stage 2) or incomplete renal recovery; (2) history of extra-renal irAEs; (3) modifiable risk factors, including baseline CKD or concomitant PPI/NSAIDs use; (4) other clinical risk factors, such as Asian race (used with caution, see below) (32), diabetes mellitus, hypertension, or genitourinary malignancy (bladder or kidney cancer) (14, 55). Modifying factors that support intermediate−risk classification include: (a) mild-to-moderate interstitial inflammation (Banff *i*-score 1–2) (67); (b) mildly elevated urinary CXCL9-to-creatinine ratio (e.g., 25–200 ng/g—a range derived from *post-hoc* analysis of data from Gupta et al. (26), where the lower bound approximates the upper interquartile range of ICI-treated patients without AKI (11 ng/g, IQR 4-23) and the upper bound approaches the diagnostic cutoff of 269.5 ng/g, though these thresholds require prospective validation; and (c) delayed initiation of glucocorticoid therapy, defined as administration beyond 3 days after AKI diagnosis (7, 8). Among patients with Banff *i*-score 1–2, the degree of interstitial inflammation is moderate but remains potentially reversible with prompt immunosuppression (61).

The high-risk category requires the presence of any core criterion: (1) severe AKI (KDIGO stage 3) or requirement for dialysis (6); (2) multi-organ irAEs involving two or more organ systems; or (3) inadequate response to glucocorticoids, defined as lack of improvement in kidney function after 3–7 days of prednisone at 0.5–1 mg/kg/day. Modifying factors that support high−risk classification include: (a) Banff *i*-score ≥2 (indicating moderate-to-severe interstitial inflammation) or concomitant glomerular pathology (61, 64); (b) presence of the *PCCA* rs16957301 risk genotype (25); (c) urinary CXCL9-to-creatinine ratio ≥269.5 ng/g (26); and (d) sIL-2Rα ≥ 1.75 × ULN (30). As with the lower tiers, the biomarker−based criteria remain hypothesis−generating.

Several specific criteria warrant additional caution. Asian race was identified as an independent predictor of ICI−AKI incidence in a single−center study (32) involving only 12 Asian patients, with a wide confidence interval (aHR 4.18–4.39, 95% CI 1.09–16.04). Its predictive value for AKI recurrence upon rechallenge is entirely unknown, and given the small sample size and lack of external validation, we do not recommend using Asian race as a standalone criterion for risk upgrading; it should prompt increased vigilance and consideration of other corroborating risk factors. KDIGO stage 3 AKI is a well−established risk factor for initial ICI−AKI (8, 19, 36), but its predictive value for recurrence after complete renal recovery has not been directly demonstrated. Patients who experienced severe but fully reversible AKI—particularly those with isolated ATIN on biopsy and no glomerular pathology—may not necessarily be at high risk for recurrence; reclassification may be warranted in such cases after multidisciplinary discussion. Finally, the biomarker−based criteria (urinary CXCL9, sIL−2Rα) are derived from diagnostic studies and have not been validated for predicting recurrence; their inclusion as modifying factors is hypothesis−generating and intended to guide future research rather than current clinical practice.

Complex or borderline cases—those in which core criteria from different risk tiers conflict (e.g., KDIGO stage 3 AKI but complete recovery, isolated ATIN on biopsy, and no glomerular disease)—require a structured approach. We recommend the following: re−evaluation after 4–6 weeks of observation with serial SCr monitoring; obtaining confirmatory biopsy if not already performed; convening multidisciplinary discussion (oncology + nephrology); and considering reclassification if renal recovery is complete and pathology shows isolated ATIN with Banff *i*−score ≤1. This approach acknowledges that no single criterion is determinative and that clinical judgment remains essential.

**Table 3** consolidates the definition criteria for the three-tier risk stratification model. The modifying factors listed for each tier are not required to be present simultaneously; rather, the presence of any single modifying factor may further support classification into that tier, provided that the core criteria are met.

### 3.2 Stepwise assessment process

The proposed model follows a hierarchical three-step framework that integrates clinical, pathological, and molecular domains. This hierarchical design ensures real-world adaptability across diverse clinical settings. In resource-limited settings such as primary or community hospitals, Step 1 (clinical assessment) alone provides a practical basis for initial risk stratification. In regional centers with biopsy capabilities, Steps 1 and 2 can be integrated. In academic or tertiary hospitals with access to advanced molecular assays, Step 3 may be applied for research purposes, though its clinical utility in predicting recurrence remains investigational.

Step 1: Clinical Baseline Assessment. All patients should undergo evaluation of AKI severity according to KDIGO criteria; assessment of concomitant medications, including PPI, NSAIDs, RAS inhibitors, and combination ICI therapy (19, 84); evaluation of extra-renal irAEs (10); consideration of Asian race (32)—acknowledging the limitations discussed in Section 3.1; assessment of other clinical risk factors, such as diabetes mellitus, hypertension, and genitourinary malignancy (bladder or kidney cancer) (14, 55); and assessment of glucocorticoid treatment response—early initiation within 3 days is associated with higher recovery rates (7, 8, 84). Additionally, a diagnosis of AKD—defined as persistent renal dysfunction persisting beyond 7 days but within 90 days of ICI initiation—should prompt extended monitoring and may warrant reclassification of risk. In a retrospective cohort study of 226 patients with metastatic solid tumors, AKD occurred in 20.4% of patients within 90 days of ICI initiation, with independent predictors including higher body surface area (OR 8.17, P = 0.03) and baseline NSAIDs use (OR 29.74, P = 0.014) (31). Of note, the AKD diagnosis identifies patients who remain at risk beyond the acute AKI episode; such patients may benefit from more frequent renal function monitoring even after initial recovery (31).

Step 2: Pathological Information Integration. For moderate-to-severe AKI (≥KDIGO stage 2) or inconclusive cases, kidney biopsy is strongly recommended (6, 7, 84). Key parameters include interstitial inflammation grade (Banff *i*-score 0-3)—low fibrosis is associated with better response, whereas higher grades correlate with increased recurrence risk (61)—and the presence of glomerular pathology requiring alternative treatment strategies (64, 66, 68). These pathological findings directly inform the confirmatory criteria for each risk tier as defined in Section 3.1. The prognostic value of the Banff *i*-score is supported by evidence linking low levels of interstitial fibrosis to favorable treatment response and improved overall survival (61). When glomerular pathology is present, alternative immunosuppressive strategies beyond corticosteroids—such as rituximab for ICI-induced glomerulonephritis—should be considered (64, 66).

Step 3: Molecular and Cellular Biomarker-Based Fine Stratification (Research use only). For patients classified as intermediate or high-risk, advanced testing may be considered for research purposes: *PCCA* genotyping for patients of European ancestry (25); urinary CXCL9-to-creatinine ratio with established cutoff values (26, 72, 73); sIL-2Rα (30); and urinary TNF-α (31). As emphasized in Section 2.4.2, these biomarkers have been validated for diagnosis of acute ICI-AKI, not for predicting recurrence upon rechallenge. Their inclusion in the assessment process is hypothesis-generating and, until prospective validation is available, their use should be limited to research settings and does not independently alter clinical risk classification. Novel composite urinary signatures, such as IL-5 plus Fas, have shown high diagnostic accuracy for ICI-AIN (74, 86). The urinary IL-5 plus Fas signature achieved an AUC of 0.94 for diagnosing ICI-AIN in a discovery cohort using high-sensitivity proteomics (74). Machine learning approaches have also shown promise: a gradient-boosting model developed by Sakuragi and colleagues continuously predicted AKI risk in ICI-treated patients (AUC 0.880) and clustered patients into four prognostically distinct groups based on predictive features (87). Patients testing positive for these molecular or cellular markers should be upgraded by one tier in the risk classification, whereas intermediate-risk patients with negative markers may be considered for downward reclassification, though this approach requires prospective validation. Other emerging markers—including composite cytokine scores, peripheral T cell dynamics, *Trm* cells, CD163+ macrophages, and PET imaging—require prospective validation before routine clinical use (78–82).

Dynamic reassessment during rechallenge is essential, as risk stratification is not a one-time event. Patients should be reassessed at three time points: T0 (pre-rechallenge), representing the initial static classification described above; T1 (first 2 weeks of rechallenge), during which a >25% SCr increase from baseline or development of grade ≥2 extra-renal irAE should prompt temporary upgrading (e.g., intermediate → high) with intensified monitoring; and T2 (monthly intervals thereafter), during which patients who remain stable for 3 months may be downgraded one tier (e.g., high → intermediate) with reduced monitoring intensity. Additionally, the occurrence of recurrent extra-renal irAEs or initiation of new lines of anticancer therapy (e.g., subsequent chemotherapy or targeted agents) should trigger repeat risk assessment, as these may alter the immune landscape and renal risk profile.

The proposed three-step framework is illustrated in **Figure 1**. Step 3, which incorporates molecular and cellular biomarkers, is reserved for research contexts pending prospective validation of its predictive utility.

### 3.3 Quantitative associations between risk strata and rechallenge outcomes

Current evidence on rechallenge outcomes derives largely from unselected populations, and no studies have yet reported outcomes stratified by the risk categories proposed in this model. Two recent meta-analyses have synthesized available data, reporting pooled recurrence rates of 14.07% (95% CI 10.26–17.89) and 18.0% (95% CI 13.2–23.9%), respectively (88, 89). The wide range observed across individual studies—from 16.5% to 44% (8, 14)—likely reflects differences in underlying patient risk profiles, underscoring the critical need for refined risk stratification. When recurrent AKI occurs, it typically manifests at a median of 10 weeks (IQR 3–17) after rechallenge (8). The lower pooled recurrence rate in the more recent meta-analysis (14.07% vs 18.0%) may reflect evolving clinical practice, including more stringent patient selection and standardized corticosteroid protocols (88).

Meta-regression analysis further revealed that patients < 65 years had a significantly lower recurrence risk upon rechallenge compared with older patients (10.6% vs 19.1%), despite having a higher incidence of initial ICI-AKI—a finding termed an “age paradox” (88). This paradox suggests that younger patients may mount a more robust immune response that is both more likely to cause initial AKI and more effectively controlled by immunosuppression, leading to lower recurrence upon rechallenge. The mechanistic basis of this age-related difference remains to be elucidated but may involve age-associated alterations in T cell repertoire diversity and regulatory T cell function (88).

Survival outcomes also vary across studies. Whereas the Canadian cohort demonstrated improved survival with rechallenge (14), an international multicenter study77 found no significant difference (8). These discordant findings suggest that the net benefit of rechallenge depends heavily on patient selection. Several factors may explain this discrepancy: the Canadian study by Blanchette and colleagues included a broader population (n = 16,425) and defined rechallenge as ICI resumption within 6 months of AKI, whereas the international study by Gupta and colleagues (n = 429) employed stricter AKI definition criteria and shorter follow-up. Differences in baseline cancer types, ICI regimens, and corticosteroid protocols may also contribute to the divergent survival outcomes (8, 14). Prospective studies with enrollment stratified by clinical, pathological, and molecular risk factors are urgently needed to quantify the benefit-risk balance across different risk strata. Based on the risk factors summarized in Sections 2.1–2.4, we hypothesize that recurrence risk would be lowest among patients meeting all low-risk criteria, highest among those with any high-risk criterion, and intermediate for the remaining patients—though these hypotheses require validation in prospective, stratified cohorts. The quantitative estimates for each tier cannot be derived from current evidence and represent a critical knowledge gap that future studies should address.

## 4 Clinical application, controversies, and future directions

### 4.1 Clinical implementation pathway

The clinical management of ICI rechallenge should be tailored to the individual patient’s risk stratum, encompassing decisions on whether to rechallenge, the intensity of monitoring, the use of prophylactic glucocorticoids, and other supportive measures. **Table 4** outlines this recommended clinical management pathway for each risk stratum.

**Table 4:** Clinical management pathway for ICI rechallenge by risk stratum.

Item	Low-risk	Intermediate-risk	High-risk
Rechallenge decision	Proceed after confirmed renal recovery (SCr ≤1.5× baseline). Guideline frameworks support rechallenge in patients with KDIGO stage 1–2 AKI with biopsy−proven ATIN and renal recovery (6, 7, 84)	May be considered after confirmed renal recovery. For KDIGO stage 2 AKI, rechallenge is possible provided recovery is achieved and biopsy confirms ATIN (6, 7)	Generally deferred unless SCr returns to ≤1.5× baseline and multidisciplinary consensus reached. For KDIGO stage 3 AKI with partial or complete recovery, rechallenge may be considered after careful risk−benefit assessment (6). For glomerular pathology (crescentic GN, vasculitis), rechallenge generally discouraged regardless of recovery (7, 64)
Monitoring	Standard: weekly SCr/eGFR for first month, then every 2 weeks for 2–3 months (7, 84). At a minimum, monitor every 2 weeks after rechallenge (6)	Intensified: twice-weekly for first month, then weekly for 3 months	Most rigorous: three times weekly for first month, then 1–2 times weekly for 3 months
Glucocorticoids	Not required; avoid nephrotoxic medications (7, 84)	No prospective data support prophylactic corticosteroids. The Chinese consensus (84) suggests low−dose prednisone (10 mg/d) may be considered during rechallenge, though evidence is graded as “uncertain.” The ASON guideline (7) does not recommend routine prophylaxis. Sprangers et al. (6) state that limited data suggest no benefit and recommend corticosteroids only for recurrent ICI−AKI. If used, shorter taper (≤28 days) may be considered (91)	No prospective data support prophylactic corticosteroids. If rechallenge is pursued despite high risk, guideline−recommended therapeutic regimens (for acute ICI−AKI) include: prednisone 0.5–1.0 mg/kg/d for AKI stage 2, or 1.0–2.0 mg/kg/d for stage 3 (or intravenous methylprednisolone 0.5–1 g/d ×3 days for patients requiring dialysis), with taper initiated after 1–4 weeks and total duration of 8–12 weeks (6, 7, 84). Predefined AKI recurrence protocol should include pulse steroids and biologic agents (infliximab, rituximab) for refractory or glomerular cases (7, 66, 93)
Other measures	Discontinue potential ATIN−inducing drugs, particularly PPI (7, 90)	Address reversible risk factors before rechallenge	Multidisciplinary tumor-nephrology discussion; prioritize non-ICI alternatives (6, 7, 84)
Special pathology note	—	—	Glomerular pathology (crescentic GN, vasculitis, membranous nephropathy): rechallenge generally discouraged; consider rituximab if rechallenge pursued (7, 64, 66)

SCr, serum creatinine; eGFR, estimated glomerular filtration rate; AKI, acute kidney injury; ASON, American Society of Onco−Nephrology; ATIN, acute tubulointerstitial nephritis; GN, glomerulonephritis; ICI, immune checkpoint inhibitor; irAE, immune−related adverse event; KDIGO, Kidney Disease: Improving Global Outcomes; PPI, proton pump inhibitor.

The low-risk category: Rechallenge can proceed after confirmed renal recovery. Standard monitoring is recommended, consisting of weekly SCr/eGFR for the first month, then every 2 weeks for 2–3 months (7). Prophylactic glucocorticoids are not required; nephrotoxic medications should be avoided (84). Discontinuation of potential ATIN-inducing drugs, particularly PPI, may further improve renal recovery (90). For these patients, recurrence risk is expected to be low based on meta-regression estimates for younger patients without major risk factors, though prospective validation is needed (88).

The intermediate-risk category: Rechallenge may be considered after confirmed renal recovery. Intensified monitoring is warranted, with twice-weekly assessments for the first month, then weekly for 3 months. No prospective data support prophylactic corticosteroids in this setting; if used, their application should be restricted to clinical trials or selected cases after multidisciplinary discussion (6, 7). Short-term prophylactic glucocorticoids (prednisone 0.5–1 mg/kg/day) have been proposed, with shorter tapers (≤ 28 days) showing comparable efficacy to longer regimens in a retrospective analysis (91). Alternatively, low-dose prednisone (10 mg/day) has been suggested as a prophylactic strategy, though the evidence is graded as “uncertain” (84). Reversible risk factors should be addressed before rechallenge. For these patients, recurrence risk is intermediate based on pooled meta-analysis estimates, though this remains a hypothesis awaiting prospective validation (88, 89).

The high-risk category: Rechallenge should generally be deferred unless SCr returns to ≤ 1.5 × baseline and multidisciplinary consensus is reached. For patients with severe glomerular pathology (e.g., crescentic GN or vasculitis), rechallenge is generally discouraged regardless of renal recovery (64, 84). Multidisciplinary tumor-nephrology discussion is essential; non-ICI alternatives should be prioritized (7, 84). No prospective data support prophylactic corticosteroids; if rechallenge is pursued despite high risk, guideline-recommended therapeutic regimens for acute ICI-AKI (prednisone 0.5–2.0 mg/kg/d, with taper over 8–12 weeks) may be considered, though these are derived from treatment of acute injury rather than prophylaxis (6, 7, 84). A predefined AKI recurrence protocol should be established, including pulse steroids (92) and biologic agents (infliximab, rituximab) for refractory cases (66, 68, 93). For patients with prior severe ICI-AKI who require rechallenge, infliximab has shown efficacy in achieving durable renal recovery in relapsed cases: in one series, 4 of 10 patients with relapsed ICI-associated ATIN achieved complete renal recovery after infliximab treatment (93). For these patients, recurrence risk is expected to be highest, though quantitative estimates remain indirect and require prospective validation.

### 4.2 Current controversies

Evidence-based rechallenge timing remains lacking. Current guidelines provide only qualitative recommendations (6, 7). Although the median time to rechallenge is approximately 1.8–1.9 months (8, 36), the optimal window remains undefined. Late-onset nephritis occurring months after ICI discontinuation suggests that different pathologies may have distinct “safety windows,” as illustrated by a case report of membranous nephropathy presenting 6 months after pembrolizumab discontinuation (94, 95). Moreover, recurrent renal injury following ICI therapy may manifest as a different pathological entity from the initial episode, as documented in a case where ATIN was followed by anti−GBM glomerulonephritis seven months after ICI initiation, with the second episode progressing to dialysis dependence (96). This case highlights that conventional monitoring windows (typically 1–3 months) may miss late-onset renal irAEs, raising the question of whether extended surveillance—particularly for glomerular pathologies—should be considered after ICI discontinuation (94). Beyond timing, the generalizability of any rechallenge framework across diverse populations is equally uncertain.

Racial differences pose another challenge to model generalizability. The incidence of ICI-AKI in Asian populations is significantly higher than in White populations (32), yet the *PCCA* risk variant has only been validated in individuals of European ancestry (25). Multi-ethnic external validation is urgently needed. Beyond the *PCCA* variant, the *HLA-DQA1* eQTL (rs3104371) identified by Kang et al. showed T cell-specific expression patterns that may vary across populations, though its association with ICI-AKI across different ancestries remains unexplored (71). Beyond genetic susceptibility, another major translation bottleneck lies in biomarker validation.

The applicability of the proposed framework to special populations remains to be defined. CKD and genitourinary malignancy are already incorporated as clinical risk factors in the intermediate-risk tier (Section 3.1 and **Table 3**) (10, 14, 19); age, although discussed in the context of the “age paradox” in recurrence risk (Section 3.3) (88), was not included as a standalone criterion due to the absence of quantitative data on its modifying effect. Importantly, all three factors were derived from studies of incident ICI−AKI rather than recurrence, and their specific impact on rechallenge outcomes remains unquantified. Until dedicated validation is available, we recommend intensified monitoring rather than automatic tier adjustment for patients with CKD or older age, and caution when applying the framework to tumor types underrepresented in the source literature.

Biomarker translation faces clinical bottlenecks that limit the predictive utility of these markers for recurrence. Although urinary CXCL9 and TNF-α show excellent diagnostic performance (26–29, 72–74), their utility for predicting recurrence risk upon rechallenge remains unexplored. Current diagnosis remains limited by the poor sensitivity and specificity of serum creatinine (20, 84, 97, 98). A key unanswered question is whether biomarker levels at the time of renal recovery—rather than at AKI diagnosis—can predict subsequent recurrence upon rechallenge. Prospective studies measuring serial CXCL9 and sIL-2Rα before and after ICI rechallenge are needed to address this gap.

The role of prophylactic glucocorticoids remains controversial. Short-term prophylaxis shows potential value, but the benefit-risk ratio lacks prospective confirmation (88, 91). Glucocorticoids may theoretically attenuate ICI antitumor responses, though retrospective data have not identified a negative association with survival (7, 20, 91). No consensus exists regarding optimal dosing, duration, or tapering schedule (7, 84). The shorter corticosteroid duration (≤ 28 days) appears safe based on a multicenter retrospective analysis (91), but whether even shorter regimens or lower initial doses provide adequate protection for intermediate-risk patients remains unknown. Randomized trials comparing prophylactic glucocorticoids versus placebo in patients undergoing rechallenge are urgently needed.

Three major guidelines provide recommendations on corticosteroid use in ICI−AKI, but these are based on therapeutic use in acute injury, not on prophylaxis during rechallenge. The Nat Rev Nephrology position paper (6)—which uses CTCAE rather than KDIGO grading—recommends prednisone 0.5–1 mg/kg/d for CTCAE grade 2 and 1–2 mg/kg/d for grade 3 renal toxicity, with tapering over 4–6 weeks. The American Society of Onco−Nephrology guideline (7) recommends prednisone 0.8–1 mg/kg/d (maximum 60–80 mg/d) for ICI−AKI, with pulse methylprednisolone 0.5–1 g/d for up to 3 days in patients requiring dialysis, a total duration of 6–8 weeks, and notes that shorter tapers (≤28 days) may be considered in select patients (91). The Chinese consensus (84) recommends prednisone 0.5–1.0 mg/kg/d for KDIGO stage 2 and 1.0–2.0 mg/kg/d for stage 3, with taper after 2–4 weeks and total duration 8–12 weeks. Importantly, the prophylactic value of these regimens is unproven: Sprangers et al. (6) state that “limited data suggest they provide no benefit” and recommend corticosteroids only for recurrent ICI−AKI; the ASON guideline (7) does not recommend routine prophylaxis; and the Chinese consensus (84) grades its low−dose prednisone (10 mg/d) suggestion as “uncertain.” Thus, prophylactic corticosteroids should be restricted to clinical trials or selected cases after multidisciplinary discussion.

Underdiagnosis of ICI-associated nephritis is an additional concern. Since a diagnosis of ATIN requires kidney biopsy, many cases may be missed (20, 34, 99). Efforts to increase the appropriate use of kidney biopsy in patients with suspected ICI-AKI are needed to improve diagnostic accuracy and guide treatment decisions. Non-invasive biomarkers such as urinary CXCL9 and sIL-2Rα may help prioritize patients for biopsy, but their negative predictive value for excluding ICI-ATIN has not been established. A diagnostic algorithm combining clinical risk scores with urinary biomarkers could potentially reduce unnecessary biopsies while maintaining diagnostic accuracy (86).

ICI-AKI may confer sustained long-term cardiovascular and kidney risks. Beyond the immediate episode, ICI-AKI may confer sustained adverse outcomes. A large cohort study demonstrated that patients with ICI-AKI had higher risks of all-cause mortality (HR 1.27), major adverse kidney events (HR 3.83), and major adverse cardiovascular events (HR 1.35) compared with those without ICI-AKI (100). These findings underscore that ICI-AKI is not merely an acute, self-limited complication but may serve as a sentinel event for heightened long-term cardiorenal risk. Whether these risks can be mitigated by optimal management of the initial AKI episode—including prompt corticosteroid initiation and avoidance of nephrotoxic medication—remains an open question requiring further study (100).

### 4.3 Future research directions

#### 4.3.1 Prospective clinical validation of the risk stratification framework

The most immediate priority is prospective, multicenter validation of the proposed three−tier framework with standardized phenotyping across diverse populations (8, 14). Stratified enrollment by clinical risk categories is essential to quantify true recurrence risk across different strata and to validate the hypothesized risk estimates presented in Section 3.3. The “age paradox”—whereby younger patients have higher ICI−AKI incidence yet lower recurrence risk—mandates age−stratified analyses to elucidate whether the framework performs differently across age groups (88). Multi−ethnic validation is equally critical, given that the *PCCA* risk variant has only been validated in European−ancestry populations (25) while Asian race has been associated with higher ICI−AKI incidence in single−center studies (32). Prospective studies should also determine the optimal rechallenge window, as current guidelines provide only qualitative recommendations (6, 7, 84). Adaptive platform trials that randomly assign patients to different rechallenge windows (e.g., 1–3 months versus 3–6 months after AKI) with stratification by risk category, using recurrent AKI as the primary endpoint, would provide the highest−level evidence to guide clinical practice.

#### 4.3.2 Development of biomarkers for predicting recurrence

While urinary CXCL9, serum sIL−2Rα, and urine TNF−α have demonstrated strong diagnostic performance for acute ICI−AKI (26, 28, 30), their utility for predicting recurrence upon rechallenge remains entirely unexplored. Prospective studies should evaluate whether pre−rechallenge levels of these biomarkers—or their dynamic trajectories during the recovery phase—can identify patients at lowest or highest risk of recurrent injury. Novel urinary signatures, such as the IL−5 plus Fas signature which achieved an AUC of 0.94 for diagnosing ICI−AIN in a discovery cohort (74), warrant dedicated evaluation in the rechallenge context. Serial monitoring of biomarker trajectories during the first weeks following rechallenge could also enable early detection of subclinical recurrent injury, potentially allowing preemptive immunosuppression before overt AKI develops (86). Notably, biomarker studies must be designed specifically for prediction rather than diagnosis, as the two applications require distinct validation frameworks. Composite cytokine scores that predict overall irAE risk (79) and urinary cytokine panels (29) may also offer insights for ICI−AKI−specific prediction, but require dedicated prospective evaluation.

#### 4.3.3 Mechanistic insights and targeted, steroid−sparing strategies

Although corticosteroids remain first-line therapy for ICI-AKI, complete renal recovery occurs in only a minority of biopsied cases (8, 61), and long-term steroid exposure increases morbidity. Future research should prioritize steroid-sparing targeted therapies. Preclinical models have identified tissue-resident memory T (Trm) cells that persist in the kidney after initial injury and orchestrate recurrent nephritis upon rechallenge (80); whether modulating these cells can prevent recurrence without compromising antitumor immunity warrants investigation. The CXCL9-CXCR3 axis and resident macrophages have also been implicated in ICI-nephrotoxicity (27), suggesting chemokine receptor blockade as a rational therapeutic target. Biologic agents have shown promise in refractory cases (66, 68, 93); whether they can be used as upfront or preventive therapies in high-risk patients undergoing rechallenge requires prospective evaluation.

#### 4.3.4 Integration of emerging technologies for dynamic risk assessment

Beyond traditional parameters, emerging technologies offer transformative potential for individualized risk stratification. Machine learning approaches have already shown promise: a gradient-boosting model continuously predicted AKI risk in ICI-treated patients (AUC 0.880) and clustered patients into prognostically distinct groups (87). Future models should integrate multimodal data—clinical variables, longitudinal biomarkers, imaging features, and genomic profiles—to enable dynamic, real-time risk prediction. Single-cell sequencing and spatial transcriptomics of kidney biopsies could characterize immune infiltrates at unprecedented resolution, identifying cell-type-specific signatures that distinguish high- from low-risk patients (21, 27). Multi-omics integration may uncover novel pathways amenable to therapeutic targeting. Research designs must also accommodate varying resource settings: academic centers should evaluate integrated multi-omics models, regional hospitals should validate clinically accessible algorithms, and primary care settings should prioritize non-invasive biomarker panels for initial triage and timely referral.

In summary, the path forward requires prospective validation of risk stratification models across diverse populations, development of biomarkers specifically designed for predicting recurrence, mechanistic exploration of targeted steroid−sparing therapies, and integration of emerging technologies for dynamic, individualized risk assessment. These efforts will transform current empirical decision−making into a precision medicine framework wherein rechallenge decisions are individualized, evidence−based, and dynamically informed by the patient’s evolving clinical, pathological, and molecular profile.
